# Correlation of t(14;18) translocation breakpoint site with clinical characteristics in follicular lymphoma

**DOI:** 10.2478/raon-2023-0030

**Published:** 2023-07-13

**Authors:** Matej Panjan, Lucka Boltezar, Srdjan Novakovic, Ira Kokovic, Barbara Jezersek Novakovic

**Affiliations:** Division of Medical Oncology, Institute of Oncology Ljubljana, Ljubljana, Slovenia; Medical Faculty Ljubljana, University of Ljubljana, Ljubljana, Slovenia; Department of Molecular Diagnostics, Institute of Oncology Ljubljana, Ljubljana, Slovenia

**Keywords:** follicular lymphoma, t(14;18) translocation, breakpoint region, clinical characteristics

## Abstract

**Background:**

t(14;18)(q32;q21) translocation is an important genetic feature of follicular lymphoma resulting in antiapoptotic B-cell lymphoma 2 (BCL2) protein overexpression. On chromosome 18 breakpoint-site variation is high but does not affect BCL2. Breakpoint most commonly occurs at major breakpoint region (MBR) but may happen at minor cluster region (mcr) and between MBR and mcr at 3′MBR and 5′mcr. The aim of this study was to analyze the correlation of t(14;18)(q32;q21) breakpoint site with clinical characteristics in follicular lymphoma.

**Patients and methods:**

We included patients diagnosed with follicular lymphoma who received at least 1 cycle of systemic treatment and had the t(14;18)(q32;q21) translocation detected by polymerase chain reaction (PCR) at MBR, mcr or 3′MBR prior to first treatment. Among patients with different breakpoints, sex, age, disease grade, stage, B-symptoms, follicular lymphoma international prognostic index (FLIPI), presence of bulky disease, progression free survival and overall survival were compared.

**Results:**

Of 84 patients, 63 had breakpoint at MBR, 17 at mcr and 4 at 3′MBR. At diagnosis, the MBR group had a significantly lower disease stage than the mcr group. Although not significant, in the MBR group we found a higher progression-free survival (PFS) and overall survival (OS), lower grade, age, FLIPI, and less B-symptoms.

**Conclusions:**

Compared to patients with mcr breakpoint, those with MBR breakpoint seem to be characterised by more favourable clinical characteristics. However, a larger study would be required to support our observation.

## Introduction

Follicular lymphoma is a low grade B-cell lymphoma, derived from germinal center. In Europe and USA, it is the second most common type of lymphoma. Follicular lymphoma is considered an incurable disease. It is characterized by an indolent clinical course though it may transform into a more malignant diffuse large B-cell lymphoma.^1^ An important genetic feature of follicular lymphoma is the translocation between the chromosomes 14 and 18, which is present in up to 90% of follicular lymphoma.^2^ The clinical significance of the translocation remains unclear as conflicting results have been reported regarding its correlation with outcome.^3,4^ Although not limited to follicular lymphoma^5^, the translocation helps in follicular lymphoma diagnosing, as well as response evaluation through minimal disease detection.^6^

The translocation places the antiapoptotic B-cell lymphoma (*BCL2*) gene next to the transcriptional enhancer of the immunoglobulin heavy chain gene (*IGH*), resulting in BCL2 protein overexpression.^7^ BCL2 protein is a member of the BCL2 family which consists of pro- and antiapoptotic proteins as well as of proteins not linked to apoptosis. It is localized in the outer mitochondrial membrane and exerts its antiapoptotic function by binding proapoptotic BCL2 family proteins such as BAX and BAC to prevent the release of cytochrome *c* from mitochondria in the intrinsic apoptosis pathway.^8^ The translocation is an early event in lymphomagenesis and although on its own likely insufficient, it plays an important role in follicular lymphoma pathogenesis. It results in extended survival of the tumor cells which may cause the accumulation of additional oncogenic genetic aberrations. Follicular lymphoma bears many chromosomal aberrations that vary in number, mostly of unknown or questionable contribution to pathogenesis.^9,10^

The t(14;18)(q32;q21) translocation was first detected by karyotypic analysis, which is at present not used for this purpose.^11^ A commonly used method for translocation detection is Flourescence In Situ Hybridisation (FISH). FISH probes bind to the entire *IgH* and *BCL2* genes thereby indiscriminately detecting translocations at various sites across the *BCL2* gene. It has close to 100% sensitivity in the t(14;18)(q32;q21) detection.^12^ Unlike with FISH, with PCR it is possible to detect the exact breakpoint site, making it indispensable for a study of clinical implications of different breakpoints. PCR is also less expensive and time consuming. However, it does have lower sensitivity of 60–70% as PCR primers identify only short DNA sections.^13^ Alternatively, multiple primers may be used to amplify and detect different breakpoints. This method has a higher sensitivity of up to 88%.^14,15^

**FIGURE 1.**
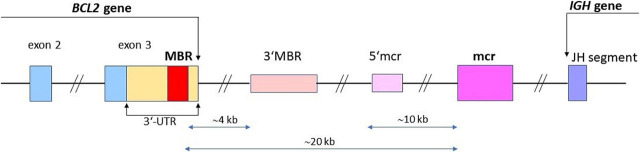
Diagram of the *BCL2/J*_H_ t(14;18) translocation breakpoints. Relative positions of major breakpoint region (MBR), 3′MBR subcluster, 5′mcr subcluster and minor cluster region (mcr) are shown according to the report of van Dongen JJM *et al*.^16^

In the t(14;18)(q32;q21) translocation, the breakpoint location on chromosome 14 is almost invariable in one of the six J_H_ gene segments, whereas on chromosome 18 different breakpoints occur relatively often. Since the breakpoint is usually located outside of the protein coding part of the *BCL2* gene, variations in the breakpoint region do not affect the *BCL2* protein. In 50% to 65% of cases the breakpoint occurs at the major breakpoint region (MBR) located at the 3′-untranslated region of the *BCL2* exon 3. In about 10–20% of cases the breakpoint occurs at the minor cluster region (mcr) located 20 kilobases (kb) from 3′ of the MBR. Additionally, the breakpoint may also be located between the MBR and the mcr, at 3′MBR and 5′mcr subclusters, commonly called the intermediate cluster region (icr).^15,16^ The 3′MBR subcluster is positioned 4 kb downstream of the MBR, while the 5′mcr subcluster is positioned 10 kb upstream of the mcr (Figure 1).^16^

The aim of this study was to analyze the correlation of t(14;18)(q32;q21) breakpoint site with clinical characteristics in follicular lymphoma.

## Patients and methods

In this clinical retrospective study, we included 84 patients diagnosed with follicular lymphoma who received at least 1 cycle of systemic treatment between 2013 and 2020 at the Institute of Oncology, Ljubljana and had t(14;18)(q32;q21) detected by PCR prior to systemic treatment. PCR was performed on bone marrow samples as a part of the diagnostic procedure. All patients included in the study signed an informed consent allowing treatment and use of their clinical information and biological material for scientific purposes. The study was approved by the Committee for Medical Ethics of Institute of Oncology Ljubljana (ERIDNPVO-0064/2022).

Data regarding treatment protocol and patients’ clinical information were collected from the clinical information system. The following characteristics observed at the time of diagnosis were gathered: gender, age, Ann Arbor stage, grade, presence of B symptoms, FLIPI score, presence of bulky disease (largest lymphoma deposit > 10 cm or mediastinal mass > 1/3 of the thoracic diameter on posterior-anterior chest x-ray), and breakpoint region of the t(14;18)(q32;q21) translocation. Progression-free survival (PFS) was defined as time from the end of the systemic treatment until relapse or end of observation, overall survival (OS) as time from diagnosis until death or end of observation and lymphoma specific OS as time from diagnosis until lymphoma-related death or end of observation. The data were collected on December 20, 2022.

DNA was isolated from bone marrow specimens using the QIAamp DNA Blood mini kit (Qiagen GmbH, Hilden, Germany). The concentration and the purity of DNA were determined using the Nanodrop spectrophotometer (Thermo Fisher Scientific, Wilmington, USA). PCR was performed using IdentiClone™ BCL2/JH Translocation Assay (*InVivo* Scribe Technologies, San Diego, CA, USA). This assay amplifies genomic DNA between primers targeting the *BCL2* gene and conserved joining regions of the *IGH* gene. Master mixes for MBR, 3′MBR and mcr detection each contained primers targeting the J region of the *IGH* gene (J_H_) and those targeting MBR, 3′MBR and mcr, respectively.

The MBR master mix contained two MBR primers (MBR1 and MBR2) and consensus J_H_ primer; the 3′MBR master mix contained four 3′MBR primers (3′MBR1-4) and consensus J_H_ primer; the mcr master mix contained three mcr primers (5′mcr, mcr1 and mcr2) and consensus J_H_ primer. Primers design and validation has been described by JJM van Dongen with colleagues.^16^ Primer sequences with National Center for Biotechnology Information (NCBI) accession numbers are shown in Table 1.

**TABLE 1.** Sequences of primers used for detection of the t(14;18)(q32;q21) translocation. Relative positions of primers are indicated downstream of the first nucleotide of corresponding reference sequence

**t(14;18) MBR primers**			

**primer name**	**NCBI accession no.**	**position**	**primer sequence**
MBR1	AY220759.1	(+193443)	5′-GACCAGCAGATTCAAATCTATGG-3′
MBR2	AY220759.1	(+192940)	5′-ACTCTGTGGCATTATTGCATTATAT-3′
**t(14;18) 3′MBR primers**			
**primer name**	**NCBI accession no.**	**position**	**primer sequence**
3′MBR1	AH010747.2	(+717)	5′-GCACCTGCTGGATACAACACTG-3′
3′MBR2	AH010747.2	(+1530)	5′-GGTGACAGAGCAAAACATGAACA-3′
3′MBR3	AH010747.2	(+1787)	5′-GTAATGACTGGGGAGCAAATCTT-3′
3′MBR4	AH010747.2	(+2718)	5′-ACTGGTTGGCGTGGTTTAGAGA-3′
**t(14;18) mcr primers**			
**primer name**	**NCBI accession no.**	**position**	**primer sequence**
mcr1	AF275873.1	(+1961)	5′-TAGAGCAAGCGCCCAATAAATA-3′
mcr2	AF275873.1	(+2407)	5′-TGAATGCCATCTCAAATCCAA-3′
5′mcr	AH010747.2	(+15849)	5′-CCTTCTGAAAGAAACGAAAGCA-3′
**Consensus J_H_ primer**			
**primer name**	**NCBI accession no.**	**position**	**primer sequence**
J_H_	OL807663.1	(+239)	3′-CCAGTGGCAGAGGAGTCCATTC-5′

AF275873.1 = homo sapiens BCL2 gene, exon 3 and breakpoint region; AH010747.2 = homo sapiens genomic sequence downstream of BCL2; AY220759.1 = homo sapiens B-cell CLL/lymphoma 2 (BCL2) gene, complete coding sequence; MBR = major breakpoint region; mcr = minor cluster region; NCBI = National Center for Biotechnology Information; OL807663.1 = homo sapiens clone J6 immunoglobulin heavy chain variable region gene, partial coding sequence

PCR products were detected by polyacrylamide gel electrophoresis (10% non-denaturing polyacrylamide TBE gel, 0.5X TBE running buffer) and visualized by UV illumination of gels stained with ethidium bromide (0.5 μg/ml). Tested samples were determined as positive for the presence of the t(14;18)(q32;q21) translocation if one or two of the amplified products (bands) within 100–2500 bp range were present. The quality of the input DNA was tested with Specimen Control Size Ladder Master Mix which targets multiple house-keeping genes and generates a series of amplicons approximately 100, 200, 300, 400, and 600 bp long to ensure control of the quality and quantity of the input DNA.

Clinical characteristics were compared among the groups defined by the breakpoint region using 1way Analysis of Variance (ANOVA) test and Independent-Samples T-test for numerical and Fisher's exact test for nominal variables. To compare OS and PFS between the groups Log Rank (Mantel-Cox) analysis was performed. p < 0.05 was defined as statistically significant.

## Results

Among 84 included patients, the group with MBR breakpoint was the most numerous with 63 patients, followed by mcr with 17 and 3′MBR with 4. Female predominance was present in all breakpoint-site groups. Overall, the median age was 61 years, with the mcr group being the oldest. Half of the 3′MBR group and up to one quarter of the 2 larger groups had grade 3 follicular lymphoma. FLIPI score was predominantly 2 or 3 and was lowest in the MBR group. B-symptoms were present in approximately half of the patients in the 3′MBR and mcr group whereas they were less common in the MBR group. Disease stage was highest in the mcr group although stage 4 was predominant in all 3 groups. Bulky disease was mostly absent in all groups with the mcr group having the lowest proportion (Tables 2,3).

**TABLE 2.** Comparison of clinical features at diagnosis between the breakpoint-site groups (MBR, 3′MBR, mcr) using Fisher's exact test

	**MBR (N = 63)**	**3′MBR (N = 4)**	**mcr (N = 17)**	**p1**	**p2**
**Male sex**	24 (38%)	1 (25%)	4 (24%)	0.571	0.391
Grade^*^ 3	11 (20%)	2 (50%)	4 (25%)	0.303	0.729
**B-symptoms**	23 (37%)	2 (50%)	8 (47%)	0.641	0.576
Bulky disease^**^	17 (27%)	1 (25%)	2 (12%)	0.497	0.335

MBR = major breakpoint region; mcr = minor cluster region; p1 = significance comparing all 3 groups; p2 = significance comparing the MBR and mcr groups only;

* = Disease grade was determined in 76 cases only;

** = Defined as largest lymphoma deposit > 10 cm or mediastinal mass > 1/3 of the thoracic diameter on posterior-anterior chest x-ray

**TABLE 3.** Comparison of clinical features at diagnosis between the breakpoint-site groups (major breakpoint region [MBR], 3′MBR, mcr)

	**MBR (N = 63)**	**3′MBR (N = 4)**	**mcr (N = 17)**	**p1**	**p2**
**Median (mean) stage**	4 (3.70)	4 (3.75)	4 (3.94)	0.361	0.023
**Median (mean) FLIPI**	2 (2.51)	3 (2.75)	3 (3.00)	0.226	0.094
**Median (mean) age**	61 (60.25)	62 (63.25)	64 (63.71)	0.423	0.218

p1 = significance comparing all 3 groups using 1 way Analysis of Variance (ANOVA) (df = 2); p2 = significance comparing the major breakpoint region (MBR) and minor cluster region (mcr) groups using Independent-Samples T-test

Comparing clinical characteristics at diagnosis, a statistically significant difference in stage was found between the MBR and mcr groups (p = 0.023). No other significant correlation was established comparing the MBR, mcr and 3′MBR groups or the 2 larger groups only (Tables 2,3).

All patients were treated with RCHOP (rituximab, cyclophosphamide, doxorubicin, vincristine, prednisolone) or RCHOP-like chemoimmunotherapy, followed by irradiation in case of residual disease. Treatment response was defined as complete remission, partial remission, stable or progressive disease, based on the positron emission tomography-computerized tomography (PetCT) 3–5 weeks after the end of systemic treatment. In case of irradiation of residual disease, additional computerized tomography (CT) was performed 3 months after irradiation and was included in final response evaluation. After systemic treatment, patients received maintenance rituximab for 2 years and were subject to a regular follow-up.

**FIGURE 2.**
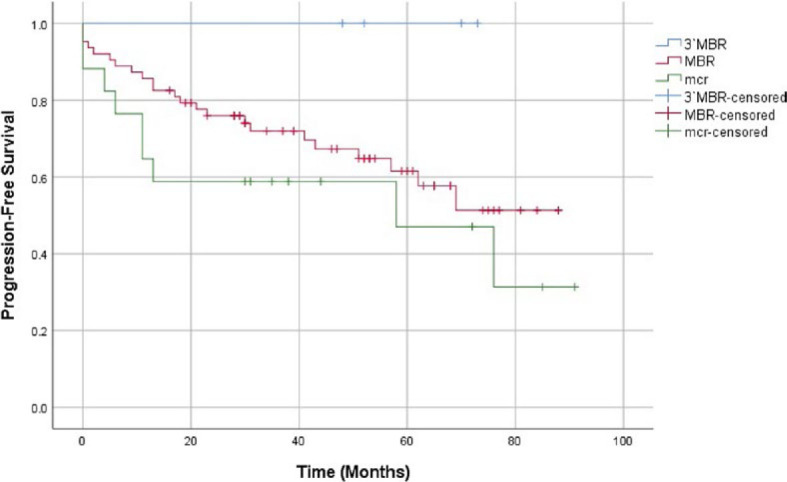
Comparison of progression-free survival between the 3′MBR (blue), MBR (red) and mcr (green) groups. Censored cases are marked as vertical lines on their respective curves. Log Rank (Mantel-Cox) significance: 0.157. Log Rank (Mantel-Cox) significance comparing the MBR and mcr group: 0.235. MBR = major breakpoint region; mcr = minor cluster region

During observation, 23 patients in the MBR and 9 patients in the mcr group relapsed and none in the 3′MBR group. The Log Rank PFS comparison found no significant difference in PFS between the 3 groups (p = 0.157) or between the 2 larger groups (p = 0.235). Though statistically insignificant, PFS was longer in the MBR group (Figure 2).

**FIGURE 3.**
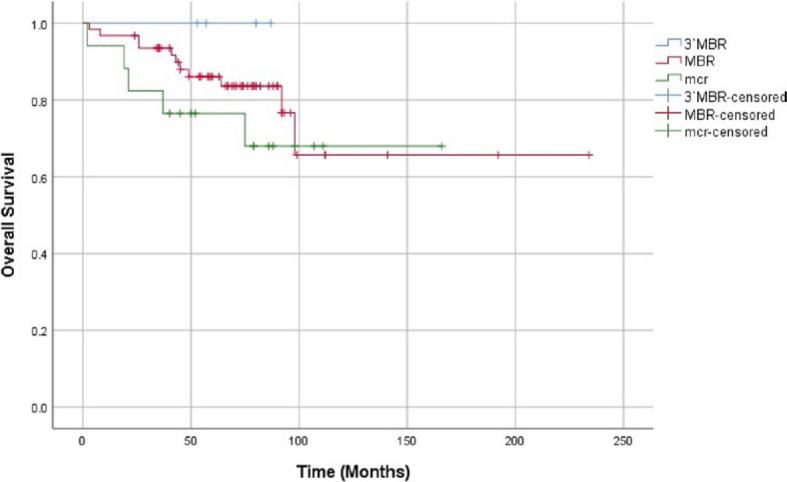
Comparison of overall survival between the 3′MBR (blue), MBR (red) and mcr (green) groups. Censored cases are marked as vertical lines on their respective curves. Log Rank (Mantel-Cox) significance: 0.426. Log Rank (Mantel-Cox) significance comparing the MBR and mcr group: 0.351. MBR = major breakpoint region; mcr = minor cluster region

**FIGURE 4.**
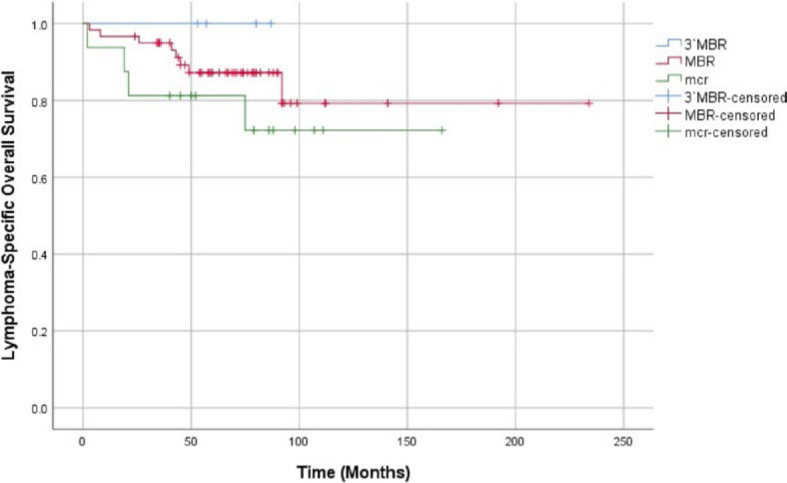
Comparison of lymphoma-specific overall survival between the 3′MBR (blue), MBR (red) and mcr (green) groups. Censored cases are marked as vertical lines on their respective curves. Log Rank (Mantel-Cox) significance: 0.409. Log Rank (Mantel-Cox) significance comparing the MBR and mcr groups: 0.301. MBR = major breakpoint region, mcr = minor cluster region

In the MBR group, 11 patients died, whereas in the mcr group the number of deceased was 5 and no patients died in the 3′MBR group. No significant difference in OS between the 3 breakpoint-site groups (p = 0.426) or the MBR and mcr group (p = 0.351) was observed (Figure 3). Lymphoma specific survival analysis yielded similar results (Figure 4).

## Discussion

It is supposed that translocation site in t(14;18) (q32;q21) translocation bears no prognostic or predictive value as it does not alter the protein-coding part of the antiapoptotic *BCL2* gene, nor does it affect *BCL2* expression level.^17^ Nevertheless, a difference in stage between the 2 common breakpoint sites mcr and MBR transpired in our routine clinical data at diagnosis, prompting this study.

Among 84 included patients, we found MBR breakpoint to be by far the most common with 63 patients, followed by mcr with 17 patients. Only 4 patients had the 3′MBR breakpoint site, making a characterisation of this group difficult.

We only found a few studies treating the subject of this article. In one of them, Weinberg *et al.* studied clinical characteristics of 236 follicular lymphoma patients with the t(14;18)(q32;q21) translocation, determining five different breakpoint regions, including MBR and mcr. MBR breakpoint was found in 118 and mcr in 11 patients.^18^ In another study, López-Guillermo *et al.* determined the *BCL2* breakpoint site in 247 patients with indolent follicular lymphoma. They determined breakpoints at the MBR and mcr regions only. MBR breakpoint was found in 175 cases and mcr in 27.^19^ Compared to the two studies, our mcr group was proportionally the largest with mcr/MBR ratio at 0.27, compared to 0.09 in Weinberg's and 0.15 in Guillermo's study.

Comparing the groups with different breakpoint region, PFS, OS and lymphoma specific OS were found to be higher in patients with MBR breakpoint site compared to mcr, though the results did not reach statistical significance. Apart from a higher proportion of bulky disease, the MBR group was indeed characterized by a more favorable disease presentation, namely lower grade, smaller proportion of patients with B-symptoms, lower FLIPI score and younger age at diagnosis. Remarkably, the MBR group also had a significantly lower clinical stage compared to the mcr (p = 0.023).

In the studies of Weinberg and López-Guillermo, no similar findings seemed to transpire. Weinberg compared MBR and “minor breakpoints” group where along with mcr, other breakpoints were included. No significant difference was found in stage, nor in age, B symptoms, FLIPI score. Furthermore, no significant difference was observed comparing PFS and OS between the two groups.^18^ López-Guillermo compared the MBR and mcr group only and found no significant difference in stage, age, gender, and B symptoms. In contrast to our finding however, he observed a significantly longer PFS in the mcr compared to the MBR group. There was only 1 relapse among 27 patients with mcr breakpoint and 42 among 175 patients with MBR breakpoint. The study of López-Guillermo was indeed performed in the setting of the low-grade follicular lymphoma, with only 3% of patients having follicular lymphoma grade 3 compared to our 22%.^19^ To obtain more relevant results for this comparison, we conducted the same comparison on our grade 1 and 2 follicular lymphoma, only to find similar results. Taken together, no clear conclusions can be drawn as to correlation between PFS and the t(14;18)(q32;q21) breakpoint region.

In conclusion, we found follicular lymphoma patients with MBR breakpoint to exhibit a more favorable clinical presentation including a higher PFS and OS. Due to our limited sample size and some incongruity in the literature, a larger study would be required to confirm our observation.
